# Progress in Electrocatalytic Hydrogen Evolution Based on Monolayer Molybdenum Disulfide

**DOI:** 10.3389/fchem.2019.00131

**Published:** 2019-03-19

**Authors:** Chuan Wang, Jinzhao Huang, Jiayue Chen, Zhongxin Xi, Xiaolong Deng

**Affiliations:** ^1^School of Physics and Technology, University of Jinan, Jinan, China; ^2^School of Mathematics and Physics, Anhui University of Technology, Ma'anshan, China

**Keywords:** monolayer MoS_2_, electrocatalytic hydrogen evolution, active sites, intrinsic catalysis, composite structure

## Abstract

Energy and environmental issues raise higher demands on the development of a sustainable energy system, and the electrocatalytic hydrogen evolution is one of the most important ways to realize this goal. Two-dimensional (2D) materials represented by molybdenum disulfide (MoS_2_) have been widely investigated as an efficient electrocatalyst for the hydrogen evolution. However, there are still some shortcomings to restrict the efficiency of MoS_2_ electrocatalyst, such as the limited numbers of active sites, lower intrinsic catalytic activity and poor interlayer conductivity. In this review, the application of monolayer MoS_2_ and its composites with 0D, 1D, and 2D nanomaterials in the electrocatalytic hydrogen evolution were discussed. On the basis of optimizing the composition and structure, the numbers of active sites, intrinsic catalytic activity, and interlayer conductivity could be significantly enhanced. In the future, the study would focus on the structure, active site, and interface characteristics, as well as the structure-activity relationship and synergetic effect. Then, the enhanced electrocatalytic activity of monolayer MoS_2_ can be achieved at the macro, nano and atomic levels, respectively. This review provides a new idea for the structural design of two-dimensional electrocatalytic materials. Meanwhile, it is of great significance to promote the study of the structure-activity relationship and mechanism in catalytic reactions.

## Introduction

**Figure 1:**
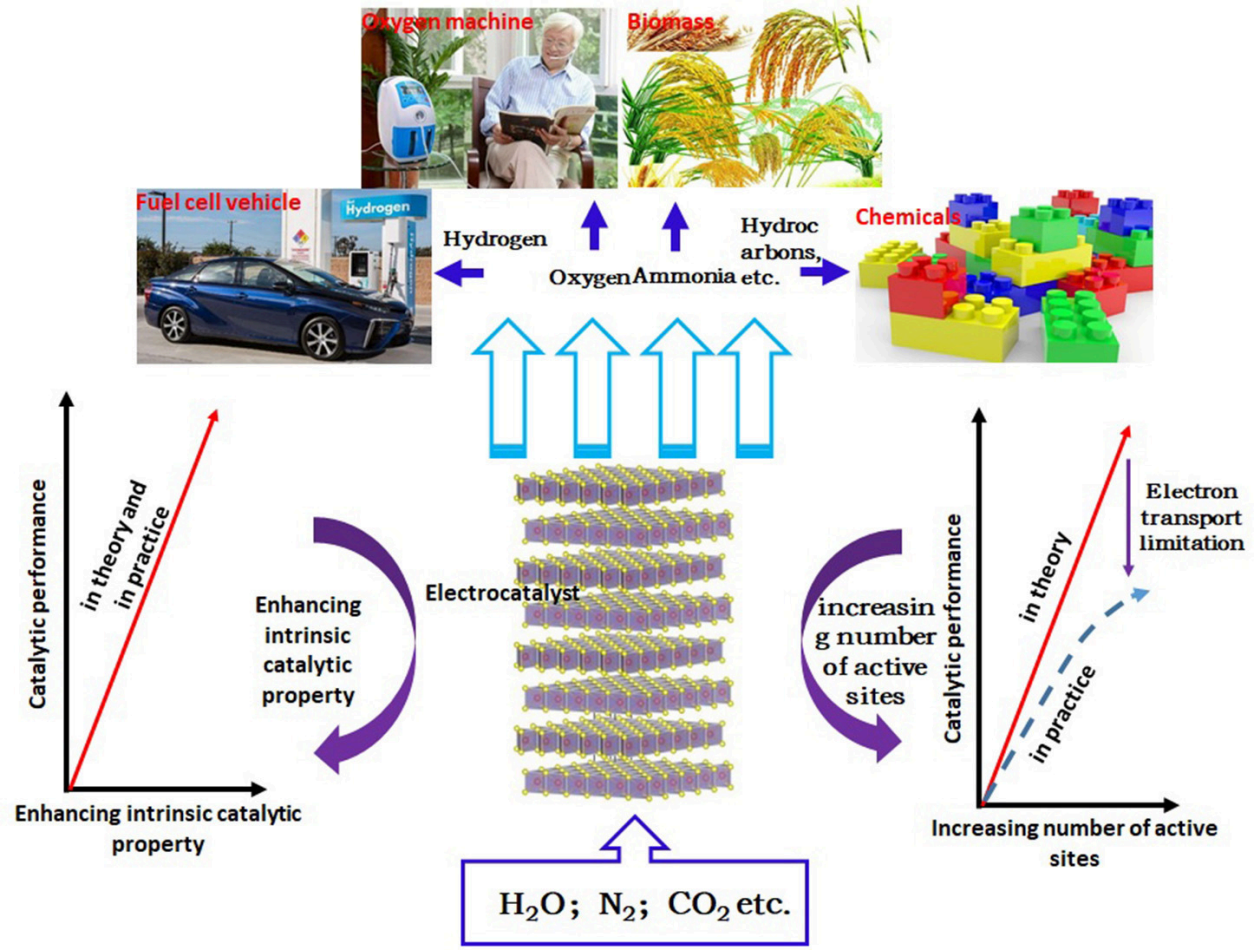
Schematic diagram of sustainable energy pattern and catalyst development strategies based on the electrocatalysis.

The continuous growth of the population and the development of the industrialization process have accelerated the consumption of fossil energy, and brought serious environmental problems. Therefore, the development of sustainable energy system is one of the most important challenges today (Wang and Mi, 2017; Chi and Yu, 2018). At present, a promising method is to produce renewable energy through the electrochemically catalytic reaction, which converts the common materials, such as water, carbon dioxide, and nitrogen, into the high-energy carriers (hydrogen, oxygen, hydrocarbons, ammonia, etc.). After years of research and practice, many important advances have been made in electrochemical energy conversion (Gu et al., 2018; Mao et al., 2018; Xiong J. et al., 2018). Among them, hydrogen energy is considered as the most powerful candidate to alternate fossil energy due to its clean, renewable, and environmentally friendly properties and high energy density (Lin et al., 2017; Zhang S. et al., 2017). Among various methods of hydrogen energy production, electrocatalytic water splitting has attracted tremendous attention because of its advantages of low cost, non-pollution and high efficiency (Wang et al., 2016). Moreover, the electrocatalytic cathode in this method is the key to determine the efficiency of water decomposition. So far, the Pt cathode possessing the near zero overpotential is considered to be the most effective catalytic cathode. However, it is difficult to be practically applied or industrialized due to its high cost and scarce resource (Eftekhari, 2017; Hou et al., 2017). Therefore, seeking for low-cost, abundant, high efficient, and environmentally friendly catalytic cathode materials has become a research hotspot. In the view of this point, many materials have been extensively explored, such as carbides, nitrides, sulfides, selenides, phosphides, and Mo-based non-noble metal electrocatalysts (Xie et al., 2014; Pu et al., 2016a,b,c, 2017, 2018; Voiry et al., 2016; Wei et al., 2016; Xie and Xie, 2016; Kou et al., 2017, 2018a,b; Jin et al., 2018). Among these materials, molybdenum disulfide (MoS_2_) has attracted much more attention due to its low cost, high catalytic activity, high stability, large in-plane carrier mobility and good mechanical properties (Tan et al., 2017; Li et al., 2018; Wang et al., 2018a). Studies have shown that monolayer MoS_2_ has higher electrocatalytic activity for hydrogen evolution. However, there are still some shortcomings, such as the limited numbers of active sites, lower intrinsic catalytic activity and poor interlayer conductivity. In order to further improve the electrocatalytic activity of monolayer MoS_2_, researchers usually composite them with other materials. In this paper, the composite of monolayer MoS_2_ with 0D, 1D, and 2D materials and its application in electrocatalytic hydrogen evolution are reviewed in order to provide guidance for related research. At present, there are two kinds of methods for preparing monolayer MoS_2_. The first method is top-down approach, including mechanical stripping (Li et al., 2012), ion intercalation (Nurdiwijayanto et al., 2018) and liquid phase stripping (Zhao et al., 2016), and the second one is bottom-up approach, including chemical vapor deposition(CVD) (Liu et al., 2018) and wet chemical stripping (Zeng et al., 2017). The development strategy of sustainable energy pattern and catalyst based on the electrocatalysis are shown in Figure 1.

## Strategies for Electrocatalytic Hydrogen Evolution

As the electrocatalyst plays an important role in improving conversion efficiency in energy conversion process, the research of electrocatalyst is a crucial part in these conversion technologies. Up to now, the electrocatalysts suffer from the lack of types and low efficiency. What's more, the high expense leads them difficult to be practically used on a large scale. Many efforts have been made to solve these problems. For example, in order to improve the electrocatalytic activity, three strategies are usually proposed: one is to increase the number of active sites (from the view of the “quantity” aspect); the other is to increase the intrinsic activity of active sites (which belongs to the “quality” aspect); the third is to improve the conductivity of electrocatalysts by forming composites. These strategies are not mutually exclusive, but can be mutually complementary to improve the activity of catalysts simultaneously (Seh et al., 2017; Tang C. et al., 2018).

Two-dimensional materials such as MoS_2_ have been extensively studied in the electrocatalytic hydrogen evolution due to their promising potential application prospect. However, there is still a big gap compared with Pt catalyst. Therefore, great efforts have been made to improve the electrocatalytic activity of MoS_2_, including phase transformation (Tang and Jiang, 2016; Jiao et al., 2018; Wang J. et al., 2018), defect engineering (Xie et al., 2013a, 2017, 2019; Xie and Yi, 2015), nanocrystallization (Yun et al., 2017), doping (Xie et al., 2013b, 2016; Sun et al., 2014, 2018; Xiong Q. et al., 2018), modification (Benson et al., 2017; Wang Q. et al., 2018) and compounding (Jayabal et al., 2017; Zhai et al., 2018), etc.

The bulk phase MoS_2_ is inert for the electrocatalytic hydrogen evolution, and the free energy of hydrogen adsorption on the base surface of MoS_2_ is 1.92 eV. However, the theoretical results show that the ΔG_H_ of Mo (1010) is 0.08 eV at 50% hydrogen coverage, which is close to the optimum value (≈0 eV) and exhibits the good electrocatalytic activity (Hinnemann et al., 2005). In addition, this propose is confirmed by the experimental results (Jaramillo et al., 2007). Theoretical and experimental studies have proved that the edge of MoS_2_ is active. Therefore, exposing more edge sites of MoS_2_ is an important method to enhance its electrocatalytic activity. Thus, the way to improve the electrocatalytic performance is classified to increase the “quantity” of active sites (Zhang J. et al., 2017). The electrocatalytic hydrogen evolution reaction is a two-electron transfer process, and the reaction rate depends largely on ΔG_H_. If the bonding between H_2_ and the surface is too weak, the adsorption (Volmer) step will limit the overall reaction rate; if the bonding is too strong, the desorption (Heyrovsky/Tafel) step will limit the reaction rate (Parsons, 1958; Wang et al., 2018b). Therefore, a highly active catalyst should have neither too strong nor too weak bonding intermediates. According to these points, by controlling the surface/interface properties of MoS_2_, the surface electronic properties, surface adsorption behavior and hydrogen evolution reaction path can be optimized, which can promote the kinetic process of the electrocatalytic hydrogen evolution and enhance the intrinsic catalytic ability (Otyepková et al., 2017; Chen et al., 2018). The research in this field is to improve the electrocatalytic activity by optimizing the “quality” of the active site. It has been proved that the transport of electrons between MoS_2_ layers needs to overcome certain barriers. The electron transport is dominated by the jump transport mode leading to the low transport efficiency, which limits the improvement of their electrocatalytic activities. So, the acceleration of the electron transport between layers is also an effective way to enhance the catalytic activity (Yu et al., 2013).

## Electrocatalytic Hydrogen Evolution

### Monolayer MoS_2_

Monolayer MoS_2_ exhibits relatively high electrocatalytic activity due to the exposure of more active sites, which can enhance the “quantity” of active sites. Zhang et al. prepared monolayer MoS_2_ by low-voltage CVD method (Shi et al., 2014). By changing the growth temperature or the distance between source and substrate, the controllable boundary length of MoS_2_ was successfully realized. The electrocatalytic hydrogen evolution results showed that the exchange current density increased linearly with the increase of boundary length. By changing the morphology of monolayer MoS_2_, the boundary length could be further extended. The dendritic morphology enriched the boundary of monolayer MoS_2_ to a great extent, which contributed greatly to the enhancement of the electrocatalytic activity (Zhang et al., 2014; Xu et al., 2018). Fractal monolayer MoS_2_ can also promote the efficiency of the electrocatalytic hydrogen evolution reaction. The fractal monolayer MoS_2_ synthesized on the surface of fused quartz can expose a large number of active sites at its edge. Besides, the existence of large internal stresses in the fractal monolayer MoS_2_, causes more electrons to migrate to the edge active sites, further improving the electrocatalytic performance (Wan et al., 2018). This study also manifests that there is a linear relationship between the electrocatalytic hydrogen evolution activity and the number of marginal active sites of MoS_2_. The inert surface of MoS_2_ can be tuned into ordered porous structure by using template. The porous structure can increase the proportion of edge atoms (the number of active sites), resulting in the enhanced electrocatalytic performance (Su et al., 2018). MoS_2_ nanosheets with rich 1T phase content can be prepared by the chemical peeling. These nanosheets possess many defects, which benefit to the good catalytic activity in the electrocatalytic hydrogen evolution (Voiry et al., 2013; Chang et al., 2016). Doping monolayer MoS_2_ can activate the activity of the base surface and improve the catalytic activity. For example, the doping of transition metal element Co atoms can change the surface electronic structure of MoS_2_ and the adsorption energy of hydrogen atoms, improving the catalytic performance (Hai et al., 2017; Lau et al., 2018). The post treatment on monolayer MoS_2_ is also a strategy to enhance catalytic capacity. Processing with oxygen plasma can increase defects and interfaces in a large extent, which play a certain role in increasing active sites and enhancing intrinsic catalytic activity (Ye et al., 2016).

However, the electrocatalytic hydrogen evolution of monolayer MoS_2_ is still limited. The number of active sites, intrinsic catalytic activity and interlayer conductance limit the further improvement of the electrocatalytic hydrogen evolution performance of monolayer MoS_2_. The process for improving the electrocatalytic properties of monolayer MoS_2_ is complicated or needs special equipments which may limit its practical application. In order to overcome the defects of monolayer MoS_2_, it is necessary to composite monolayer MoS_2_ with other low-dimensional materials.

### Composition With 0D Materials

The electronic structure of the surface and the binding energy of the active intermediates can be modulated by compositing the single layer MoS_2_ and 0D, 1D, and 2D materials, leading to the improvement of the electrocatalytic performance by means of the active sites on the “quality” aspect. At the same time, the interlayer conductance of MoS_2_ can be enhanced by forming the composite, which further improves the electrocatalytic activity from another aspect. Zhang et al. composited Pd, Pt, and Ag nanoparticles with monolayer MoS_2_ by wet chemical method (Huang et al., 2013). The Pt nanoparticles with the size of 1-3 nm are successfully composited on the surface of monolayer MoS_2_. The Pt-modified monolayer MoS_2_ showed the excellent electrocatalytic performance with the neglected overpotential and the comparable Tafel slope of 40 mV/dec compared with pure MoS_2_ and Pt, which could be ascribed to the effective collection and transport of electrons in the presence of Pt.

**Figure 2:**
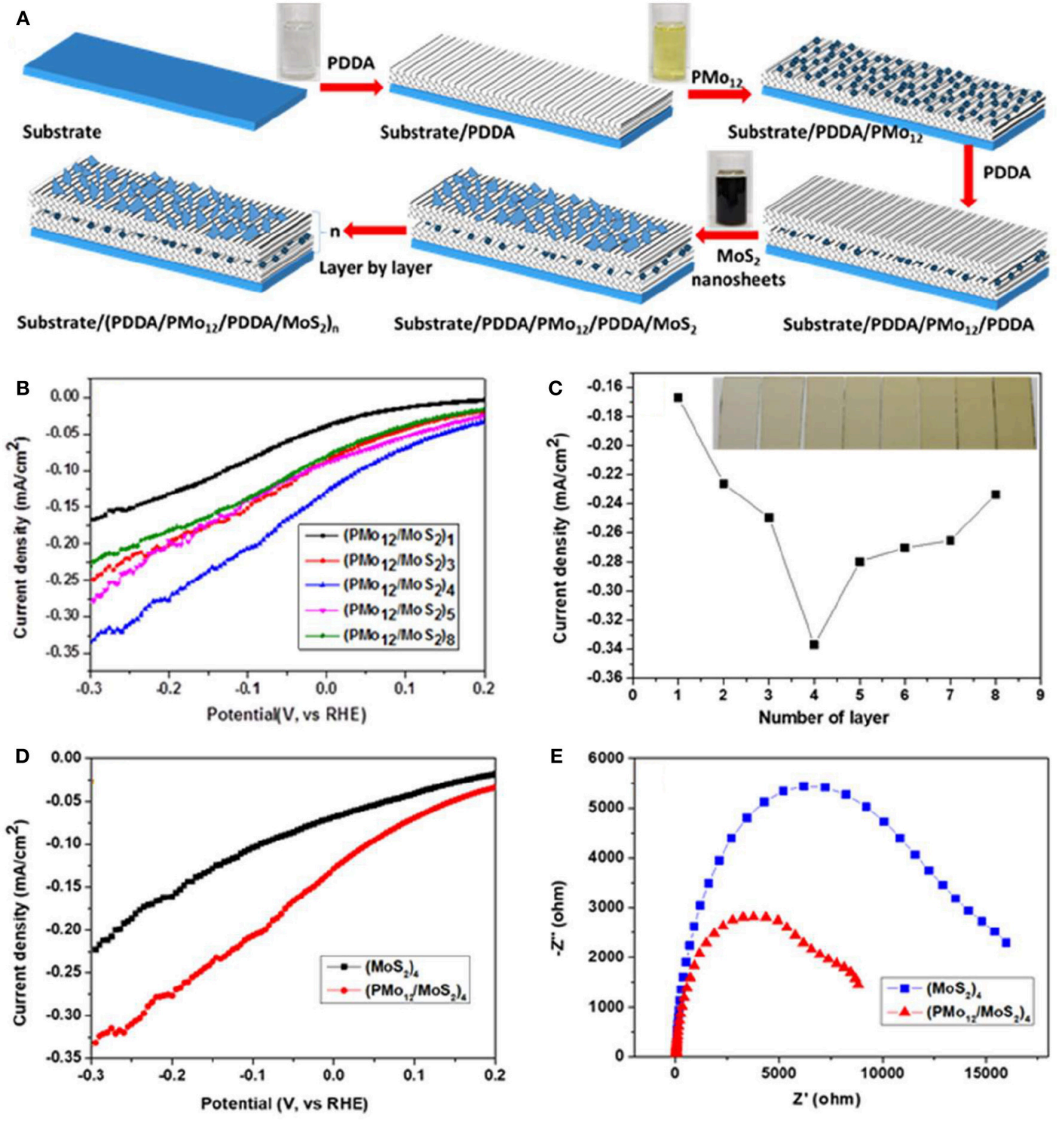
**(A)** Process for building up the multilayer films (PMo_12_/MoS_2_)_n_, **(B)** Polarization curves of multilayer (PMo_12_/MoS_2_)_n_, **(C)** Current density as a function of layer number (inset of (c) Photograph of thin films of (PMo_12_/MoS_2_)_n_ deposited on ITO with different number of layer), **(D)** Polarization curves of multilayer (PMo_12_/MoS_2_)_4_ and (MoS_2_)_4_, **(E)** The EIS spectra of multilayer (PMo_12_/MoS_2_)_4_ and (MoS_2_)_4_ (Huang et al., 2018a).

Polyoxometallates (POMs) possess excellent performance in catalysis, which is attributed to the abundant oxygen on the surfaces and rich negative charges (Huang J. et al., 2017; Huang et al., 2018b). Polyoxometallates have the abilities to accept multiple electrons and reversible redox properties, which means that they have the outstanding electronic transport properties (Ammam, 2013). The MoS_2_ nanosheets were successfully exfoliated using the liquid phase exfoliation method assisted by formamide solvothermal treatment (Huang et al., 2018a). The monolayer MoS_2_ and POM were stacked into a multilayer heterostructure by the layer-by-layer (LBL) method, and the process for buildup of multilayer films is shown in Figure 2A. The electrocatalytic performance was improved due to the high electron transport performance of POMs and the electrochemical test results were plotted in Figures 2B–E.

### Composition With 1D Materials

**Figure 3:**
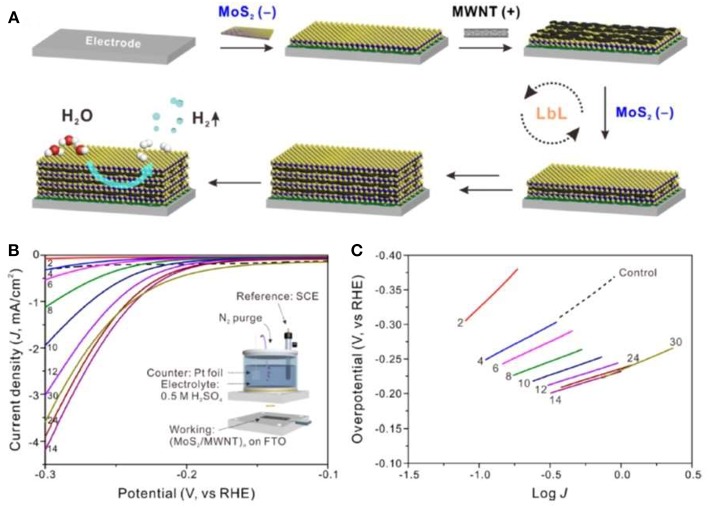
**(A)** Schematic of the LbL assembly of (MoS_2_/MWNT)_n_ multilayer electrode, **(B)** Polarization curves of hybrid multilayer (MoS_2_/MWNT)_n_ electrodes (Inset of **B** is the experimental setup of the three-electrode system), **(C)** Corresponding Tafel plots (Ahn and Kim, 2017).

One-dimensional (1D) nanostructures offer the unique electronic transport channels. By compositing them with monolayer MoS_2_, the carrier transport capacity can be improved and the carrier recombination can be reduced. At the same time, the composite structure can bring the modification of the interface and the change of electronic structure, then the electrocatalytic performance can be further improved. Kim group prepared monolayer MoS_2_ by Li intercalation method (Ahn and Kim, 2017). 1D carbon nanotubes and MoS_2_ nanosheets were composited by LBL method to form a multilayer structure. The fabrication process was shown in Figure 3A. The hydrogen evolution performance reached the optimum value with the Tafel slope of 62.7 mV/dec for the number of layers of 14. The enhanced catalytic performance was attributed to the high conductivity of carbon nanotubes, which increased the conductivity of interlayer of MoS_2_, as shown in Figures 3B,C.

Xia et al. combined Au nanorods with MoS_2_ to achieve surface plasmon resonance under auxiliary illumination, which increased the carrier concentration. Moreover, the improved carrier injection and carrier separation efficiency benefited from 1D structure can enhance the electrocatalytic efficiency (Shi et al., 2015).

### Composition With 2D Materials

The calculation results pointed out that the combination of graphene oxide and MoS_2_ could change the interface electronic structure, improve electron transport, and enhance electrocatalytic performance (Tang S. et al., 2018). The combination of graphene and monolayer MoS_2_ could increase the number of active sites, accelerate the desorption rate of H_2_ and enhance the efficiency of electron injection, and thus greatly boosted the electrocatalytic activity (Huang H. et al., 2017).

**Figure 4:**
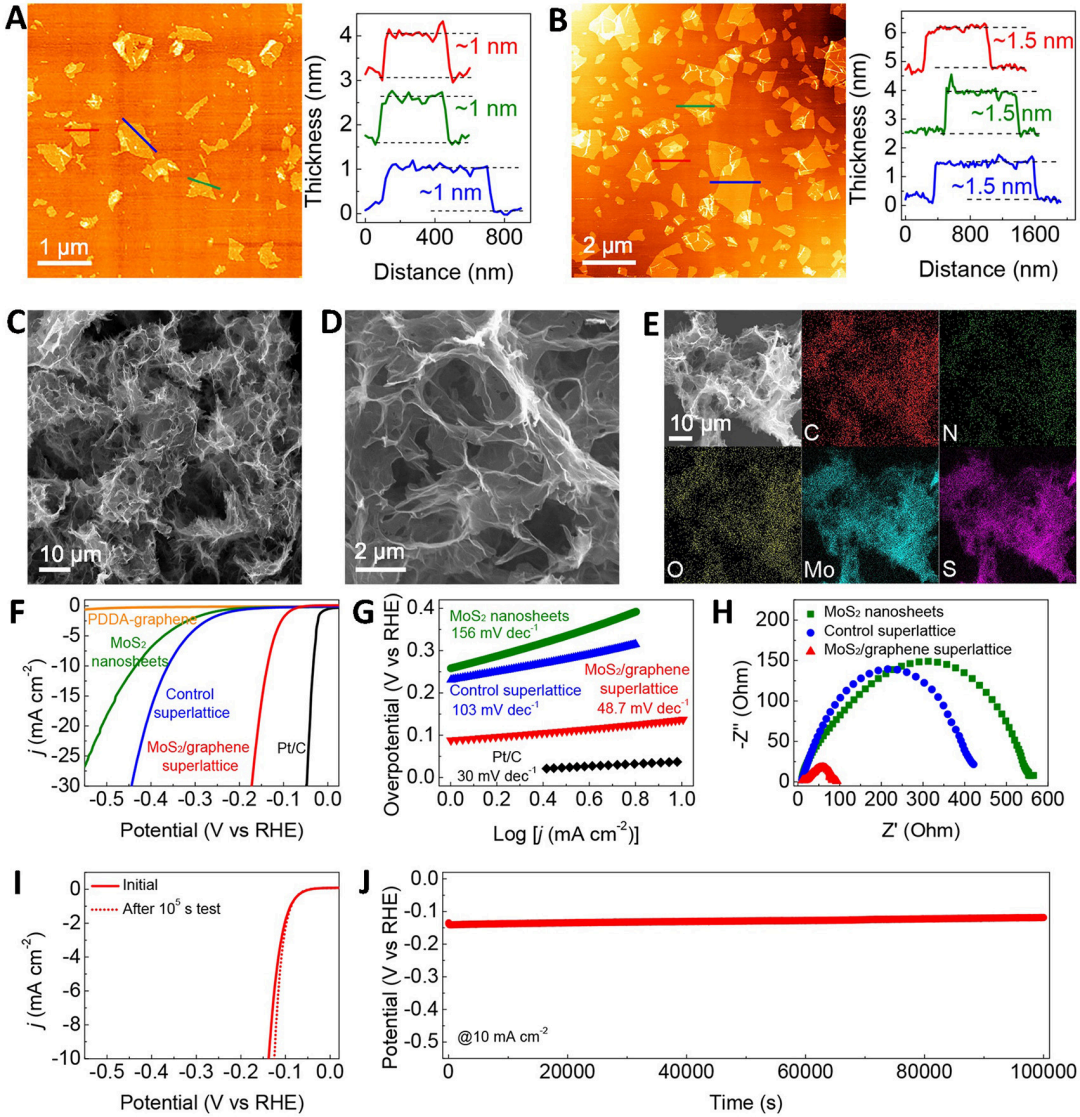
**(A)** AFM images and height profiles for the exfoliated metallic MoS_2_ nanosheets, **(B)** AFM images and height profiles for the PDDA-graphene nanosheets, **(C,D)** SEM images of the MoS_2_/graphene superlattice with different magnifications, **(E)** SEM image and corresponding elemental mapping images of the MoS_2_/graphene, **(F)** Polarization curves, **(G)** Tafel plots, **(H)** The EIS spectra, **(I)** The polarization curves of the MoS_2_/graphene superlattice before and after the 10^5^ s test, **(J)** Long-term stability measurement (Xiong P. et al., 2018).

Sasaki team successfully exfoliated bulk MoS_2_ to obtain monolayer by Li intercalation method, and the monolayer MoS_2_ was verified to be 1T phase structure (Xiong P. et al., 2018). Then, it can be seen that the monolayer MoS_2_ was successfully restacked with graphene to form composite structure by the flocculation method (Figures 4A–E). The electrochemical measurements (Figures 4F–J) showed that this structure exhibited excellent electrocatalytic hydrogen evolution performance with the overpotential of 88 mV and Tafel slope of 48.7 mV/dec. The long-term stability was also manifested at 10 mA/cm^2^ for 10,00,00 s. The outstanding electrochemical properties could be originated from the enhanced electron transport and reduced Gibbs free energy of this unique structure.

According to the characteristics of 0D, 1D, 2D materials, it can play different roles in the composite structure, which can serve as an enhanced electron transport function, as well as to increase the active site, or to activate the in-plane properties. In the actual operation, the electrocatalytic performance can be improved with diverse composite structure in different aspects, such as intrinsic catalysis, increasing the number of active sites, and improving conductivity.

## Outlook

Monolayer MoS_2_ has attracted extensive attention for the electrocatalytic hydrogen evolution. In order to overcome the limitations of active sites, low intrinsic catalytic activity and poor interlayer conductivity, surface modification and composite structure are carried out to improve the electrocatalytic performance. However, there are still some challenges to be worthy of further investigation. Firstly, the properties of composite structure, active site and interface of composite materials are not clear, and need to be studied by more detailed characterization methods; secondly, the comprehensive utilization of monolayer MoS_2_ and its composite structures at the macro, nano and atomic levels will improve the efficiency of the electrocatalytic hydrogen evolution in principal, but involving the preparation, test and mechanism explanation of materials and devices. It belongs to the multi-disciplinary frontier field and can be studied through in-depth research. In-depth research on these issues can provide the clue for the improvement of the efficiency of the electrocatalytic hydrogen evolution and the deep insight of catalytic mechanism.

## Author Contributions

All authors listed have made a substantial, direct and intellectual contribution to the work, and approved it for publication.

### Conflict of Interest Statement

The authors declare that the research was conducted in the absence of any commercial or financial relationships that could be construed as a potential conflict of interest.
